# Starting up a cementless Oxford medial unicompartmental knee arthroplasty practice: a prospective cohort study of 200 knees

**DOI:** 10.1007/s00402-026-06229-z

**Published:** 2026-02-26

**Authors:** Annika Gottholt Hansen, Kristine Ifigenia Bunyoz, Cecilie Henkel, Mette Mikkelsen, Kirill Gromov, Anders Troelsen

**Affiliations:** 1 Copenhagen University Hospital Hvidovre, Hvidovre, Denmark; 2 Faculty of Health and Medical Sciences, University of Copenhagen, Copenhagen, Denmark; 3 Department of Clinical Medicine, University of Copenhagen, Copenhagen, Denmark

**Keywords:** Knee replacement, Medial unicompartmental knee arthroplasty, Learning curve, Patient-reported outcomes

## Abstract

**Introduction:**

Medial unicompartmental knee arthroplasty is widely used to treat anteromedial osteoarthritis, yet limited data exist on outcomes during its implementation phase. Therefore, this study aimed to evaluate the learning curve and the clinical and radiological outcomes during the early implementation of medial unicompartmental knee arthroplasty.

**Materials and methods:**

The first 200 medial unicompartmental knee arthroplasty procedures performed by two arthroplasty surgeons were analyzed to assess the relationship between outcomes and the cumulative number of cases. The primary outcome was the learning curve for the duration of surgery, while secondary outcomes included the Oxford Knee Score, the Forgotten Joint Score, and the Activity and Participation Questionnaire, which were assessed at 3, 12, and 24 months postoperatively. Implant survival and positioning were evaluated at the final follow-up.

**Results:**

Cumulative summation analysis showed a data-driven reduction in surgical duration after 55 cases. Median Oxford Knee Score was 41 (IQR 34–44) at 12 months and 42 (IQR 35–45) at 24 months. Implant survival at 5 years was 97.4% (95% CI: 95.1–99.7). Radiographically, 86.5% of patients had tibial implant valgus/varus within 5° of neutral, and no tibial implant overhang exceeded 2 mm.

**Conclusion:**

Medial unicompartmental knee arthroplasty was associated with favorable clinical outcomes during early implementation. Surgical duration indicated a learning curve over 55 cases. Patient-reported outcome measures remained stable, showing reliable outcomes regardless of the learning phase. Surgical precision was maintained throughout, indicating proficient surgical outcomes even during the early phase.

**Supplementary Information:**

The online version contains supplementary material available at 10.1007/s00402-026-06229-z.

## Introduction

Unicompartmental knee arthroplasty (UKA) is an established treatment for patients with end-stage unicompartmental osteoarthritis of the knee [1, 2]. Compared to total knee arthroplasty (TKA), medial UKA (mUKA) has been associated with superior patient-reported outcomes (PROMs) [3], lower mortality [2, 4], shorter hospital stays [5], faster recovery [6], and fewer perioperative complications and readmissions [2, 7]. However, despite these advantages, UKA has been associated with a higher risk of revision compared to TKA [2], particularly in low-volume centers [8, 9] and among less experienced surgeons [10]. Reported revision risk after UKA remains debated and may be influenced by surgical usage [11] and by revision bias [12], as UKA is often revised at a lower threshold than total knee arthroplasty. Current guidelines recommend UKA as a suitable alternative to TKA in appropriately selected patients and specifically state that all knee arthroplasty surgeons should be adequately trained in the mUKA procedure [13]. Given the natural learning curve associated with the introduction of new surgical procedures [10, 14, 15], it is crucial to assess perioperative efficiency and early postoperative outcomes during the initial phase to better understand their development and impact on early results. Surgical duration is a well-established surgical process variable for assessing learning curves and reflects progressive improvements in technical performance with increasing experience. Compared with revision-based outcomes, which are infrequent, multifactorial, and influenced by long-term follow-up [16], surgical duration allows assessment of early learning effects, perioperative efficiency and workflow. Although mUKA demonstrates excellent long-term results in high-volume, experienced centres [8, 9, 17], it remains less clear how perioperative performance and early clinical outcomes evolve during the initial implementation phase, and whether implementation-phase outcomes differ from those reported in mature UKA practice. Therefore, this study aimed to investigate the learning curve for surgical duration and evaluate both clinical and radiological outcomes during the early phase of mUKA implementation as two orthopedic surgeons adopted the cementless, medial Oxford UKA with Microplasty instrumentation into clinical practice.

## Methods

### Study population

This study was based on prospectively collected data from the first 200 mUKA performed by two arthroplasty surgeons at a single center. Each surgeon performed their first 100 mUKA procedures during the study period from 2016 to 2019. The yearly usage rates for mUKA during the study period varied between 47.6 and 68.5% for surgeon AT and between 14.3 and 49.6% for surgeon KG. The total number of primary knee arthroplasties performed during the study period was 206 for AT and 301 for KG. All patients underwent a standardized preoperative rehabilitation program, which included physiotherapist-led training, as part of the standard treatment before being offered surgery. The Oxford mUKA procedure was performed using a minimally invasive technique [18] with the application of a tourniquet in all cases and involved the use of uncemented, mobile bearing implants, along with microplasty instrumentation in all cases. All patients underwent clinical evaluation by one of the arthroplasty surgeons and were selected based on the indications for mUKA proposed by the Oxford group [18]. Written informed consent was obtained from each patient. Patient-related data were collected using a standardized questionnaire administered before surgery. Basic demographic data including age, sex, body mass index (BMI), and American Society of Anesthesiologists’ physical status classification system (ASA) score, were collected.

### Statistical analysis

Normality was assessed through quantile-quantile plots and histograms. Categorical data were presented as n (%). Normally distributed continuous data were presented as mean ± standard deviation (SD), and non-normally distributed data were presented as median and interquartile range (IQR). To assess performance, a cumulative sum (CUSUM) analysis was applied to combined data from both surgeons to provide a quantitative evaluation of the learning curve for surgical duration across the first 200 mUKA procedures, reflecting implementation at the institutional level rather than surgeon-specific performance. Cases were listed chronologically, and deviations of individual surgical durations from the cohort mean were calculated. By summing these cumulative differences, a CUSUM plot was generated. Turning points were defined as changes in the slope of the CUSUM curve, identified through visual inspection and examination of the underlying cumulative values. The analysis was performed without risk adjustment to allow a simple and transparent description of temporal changes in operative performance. A sensitivity analysis was performed by comparing surgical duration between patients operated during the learning phase and those operated during the mastery phase. Statistical analyses were performed using RStudio version 4.2.3.

### PROMs

Patient-reported outcome measures (PROMs) included the Oxford Knee Score (OKS), Forgotten Joint Score (FJS), and Activity & Participation Questionnaire (APQ). The OKS was assessed preoperatively, while all PROMs were evaluated at 3, 12, and 24 months postoperatively. Non-responders to follow-up questionnaires were contacted for structured interviews to assess PROMs. Analyses of PROMs were performed using complete-case data at each follow-up time point, with patients lacking PROM responses excluded from the respective analyses. Baseline characteristics of responders and non-responders were compared to assess potential selection bias. No formal adjustment for multiple comparisons was applied, as PROM analyses were based on predefined outcome measures and were primarily descriptive rather than intended for confirmatory hypothesis testing. 12 months OKS outcomes were categorized into four groups: poor, fair, good, and excellent, based on the classification suggested by Kalairajah et al. [19, 20] (Table 1). The minimal clinically significant difference in OKS was set at 4 points, as established by Beard et al. [21].

**Table 1:** Oxford knee score classification

Category	Oxford Knee Score
Excellent	42 to 48
Good	34 to 41
Fair	27 to 33
Poor	< 27

### Implant survival, reoperations, and mortality

Kaplan-Meier survival analysis was used to determine the 5-year implant survival rate. The analysis was performed with the endpoint revision for any reason, defined as the removal or replacement of at least one component. Reoperation was defined as any surgery in the knee containing the mUKA without the removal or replacement of a component. Information regarding revisions, reoperations, and death was extracted from medical records.

### Radiographic evaluation

All patients underwent standard preoperative and postoperative short leg-length radiographs in both anteroposterior (AP) and lateral views of the knee. The radiographs were retrieved from IMPAX, anonymized, analyzed, and measured using Mdesk. All measurements were compared to the standard radiographic criteria from the surgical guideline for the Oxford mUKA [18]. Short leg-length radiographs were used as they reflect routine clinical practice at our institution. Due to the unavailability of full-leg-length radiographs, the Hip–Knee–Ankle (HKA) angle could not be assessed. Instead, the anatomical Femorotibial Angle (FTA) was measured. On the AP view, this was done by drawing lines through the mid-diaphyseal points of the femur and tibia at two levels, on the lateral view, lines were drawn along the femoral and tibial cortexes [22, 23]. FTA was measured relative to a mechanical axis of 180°, with > 180° classified as varus and < 180° as valgus. Although a healthy knee typically presents with 5–7° of physiological valgus (i.e., FTA ≈ 173–175°), no correction was made for this. This approach allowed for standardized pre- and postoperative comparisons, focusing on correction magnitude rather than absolute alignment. Illustrative examples of these measurements can be found in online resource SI2. Inter-observer reliability was not assessed, as all radiographic measurements were performed by a single observer using a standardized measurement protocol. Intra-observer reliability was not formally quantified, but measurements were repeated to minimize random measurement error.

### Ethics

Ethical approval was not required for this observational study. The PROM register was approved by the Danish Data Protection Agency (P-2022-290), and data access was granted by the hospital board of directors as part of a local quality assurance project.

## Results

### Demographic information

The mean follow-up was 5.8 (SD 1.1) years. Mean age at the time of surgery was 64.8 (SD 10.4) years, and mean BMI was 30.7 (SD 6.1). There were 89 patients (44.5%) who were male. The median preoperative OKS was 23 (IQR 19–29). The preoperative mean FTA was 182° (SD 3.34) of varus and the mean posterior tibial slope was 5.6° (SD 2.4) (Table 2). A comparison of the demographic characteristics between responders and non-responders revealed that non-responders were younger (*p* = 0.043). No significant differences were observed with respect to sex, BMI, ASA score, or follow-up time (Online resource SI1 - Table 1).

**Table 2:** Patient demographics and preoperative radiographic evaluation

Patient demographics	
Patients (knees)	200
Left knee	104
Right knee	96
Bilateral*	10
Number of males, n (%)	89 (44.5%)
Mean age (years) (SD)	64.8 (10.4)
Mean body mass index (kg/m2) *n* = 199 (SD)	30.7 (6.1)
*ASA score, n (%)*	
ASA 1	33 (16.5%)
ASA 2	131 (65.5%)
ASA 3	36 (18.0%)
Median OKS pre-surgery (IQR), *n* = 160	23 (19–29)
Mean follow-up years (SD) [range]	5.8 (1.1) [0.15–7.75]
*Preoperative alignment, mean (SD)*	
FTA	182° (3.3)
MPTA	85.7° (2.4)
JLCA	84.9° (2.0)
PTS	5.6° (2.4)
Bone on Bone, n (%)	194 (97)

* Each knee performed separately on different dates

Categorical data are displayed as *n* (%). Continuous data are summarized as mean and standard deviation (SD) or median and interquartile range (IQR), depending on normality. *BMI* body mass index (kg/m^2^), *ASA score* American Society of Anesthesiologists physical status classification, *FTA* anatomical femorotibial angle, *MPTA* medial proximal tibial angle, *JLCA* joint line convergence angle, *PTS* posterior tibial slope

### CUSUM analysis

**Fig. 1:**
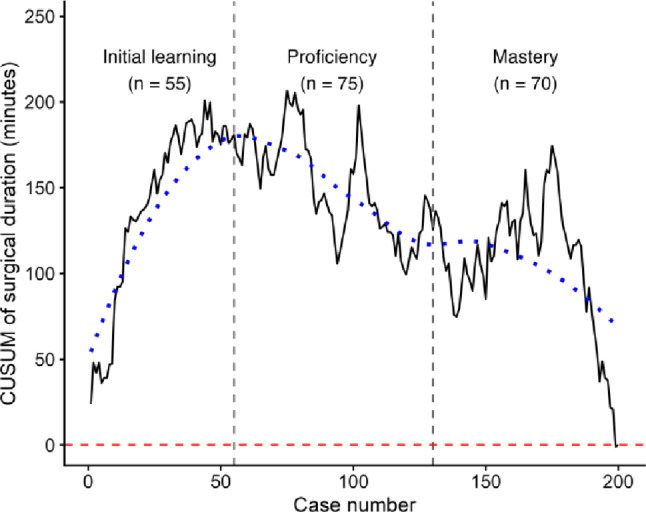
Cumulative Sum (CUSUM) analysis of surgical duration. The graph depicts the cumulative summation (CUSUM) of surgical duration across consecutive procedures, with the X-axis representing the pooled number of cases and the Y-axis indicating the CUSUM score for surgical duration. The black solid line illustrates performance trends over time, while the blue dotted line provides a smoothed representation of the overall trend. Key transition points are marked by vertical dotted gray lines: the first, at the 55th case, denotes the peak CUSUM value, corresponding to the highest cumulative surgical duration, while the second, at the 130th case, indicates the point where performance begins to stabilize. The mean surgical duration for both surgeons was 61.7 min (SD 11.7)

The overall mean duration of surgery was 61.7 (SD 11.7) minutes. The learning curve for surgical duration was divided into three phases, marked by two turning points. The first turning point, around case 55, signified the transition from the initial learning phase, characterized by longer surgical durations, to the proficiency phase, where surgical duration began to decrease. The second turning point, around case 130, marked the shift from the proficiency phase to the mastery phase, which was illustrated by a slight increase in surgical duration (Fig. 1). Mean surgical duration decreased from 65.3 (SD 10.7) minutes in the first 55 procedures (Initial learning phase) to 60.2 (SD 13.1) minutes in the last 70 procedures (Mastery phase), corresponding to a relative reduction of approximately 7.8%. This finding supports the learning-curve pattern observed in the CUSUM analysis.

### PROM scores

**Fig. 2:**
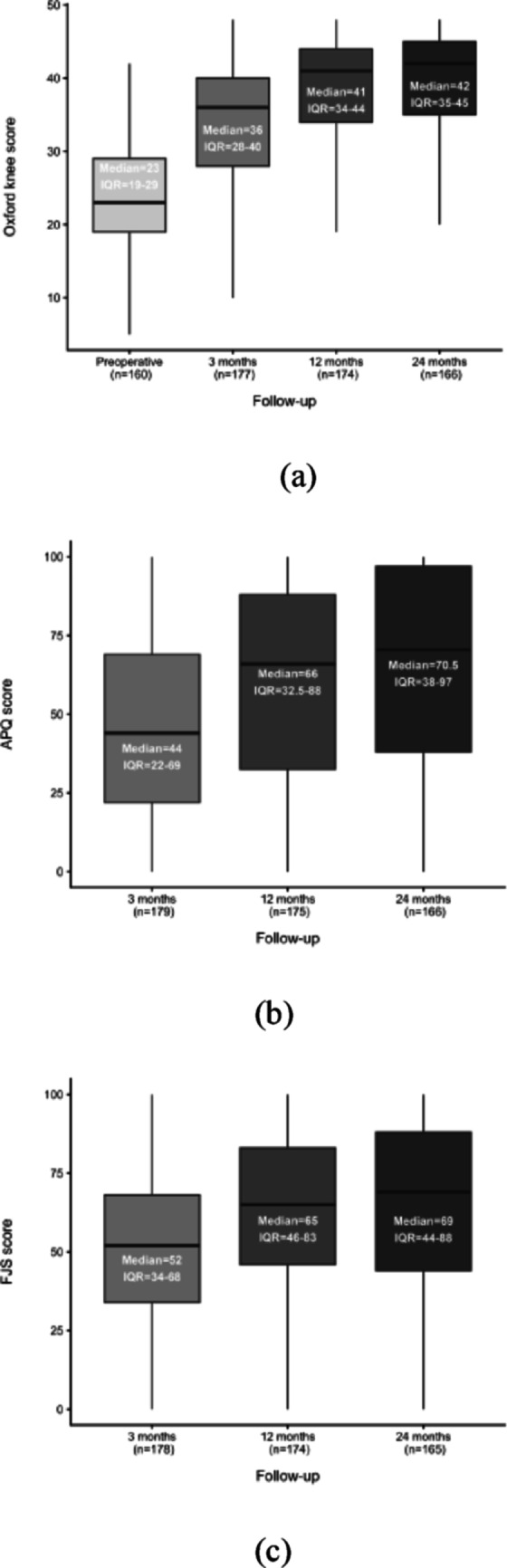
Patient-reported outcome measures (PROMs) after medial unicompartmental knee arthroplasty. Boxplots illustrating the progression in the Oxford Knee Score (**a**), the Activity and Participation Questionnaire (**b**), and the Forgotten Joint Score (**c**) over time. Scores range from 0–48 (OKS) and 0–100 (APQ, FJS). *n* represents the number of patients available for follow-up. Each box displays the median and interquartile range (IQR) of the patient-reported outcome measures (PROMs)

All PROM scores showed substantial improvement from the baseline at 3, 12 and 24 months. (Fig. 2a, b, c), (Online resource SI1, Table 2).

### Excellent vs. poor OKS

At the 12-month follow-up, responses were received from 174 patients (87%), with 79 (45.4%) reporting excellent outcomes (OKS > 41) and 27 (15.5%) reporting poor outcomes (OKS < 27). Patients with poor outcomes, compared with those with excellent outcomes, were younger on average (mean age 60.3 vs. 67.6 years, *p* = 0.01) and had a higher ASA score, with significant differences observed for ASA 2 (*p* = 0.048) and ASA 3 (*p* = 0.040). These findings are consistent with Table 3, where patients with poor outcomes (OKS < 27) are compared with all remaining patients (OKS$\:\:\ge\:$ 27), showing the same overall trend for age and ASA score. At the 24-month follow-up, responses were received from 166 patients (83%), with 90 (54.2%) reporting excellent OKS and 21 (12.7%) reporting poor OKS.

### The learning curve’s effect on OKS outcome

When dividing the 12-month OKS scores chronologically into four groups, no notable differences were observed over time, despite varying levels of experience, as all group scores differed by less than 4 points. The median OKS scores were 41.0 in the first group, 41.5 in the second group, 41.0 in the third group, and 39.5 in the fourth group. Across all groups, the median OKS remained within the good or excellent category.

### Reoperation, revision, and death

**Fig. 3:**
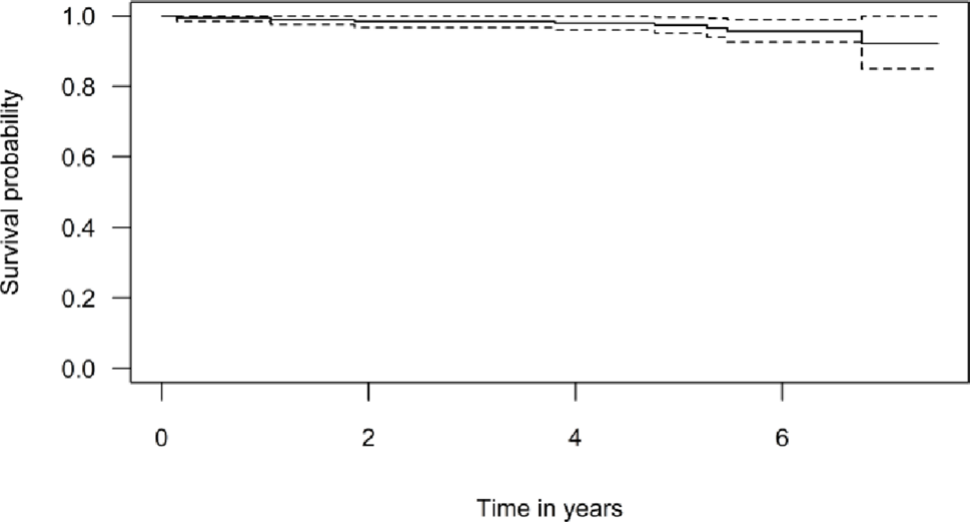
Kaplan-Meier graph with implant revision as endpoint. Kaplan-Meier survival graph with “implant revision” as the endpoint, estimating a 97.4% implant survival at 5 years. The solid black line represents the estimated survival, while the dashed lines indicate the 95% confidence interval.

Six knees (3.0%) underwent reoperation, with a mean time to reoperation of 4.0 years. The causes were impingement (2/6), reduced range of motion (1/6), and lateral progression of osteoarthritis (OA) (3/6). Eight knees (4.0%) underwent revision surgery, with a mean time to revision of 3.6 years. The indications for revision were progression of OA (5/8), pain (1/8), and bearing dislocation (2/8). One patient underwent re-operation for impingement in 2019 and subsequently underwent TKA in 2021 due to pain. Revision events were mainly observed in the early to mid-phase of the learning curve, whereas reoperations were more evenly distributed across the course of cases, including the late phase (Online resource SI1 - Table 3; Fig. 1). Mean follow-up was 5.8 (SD 1.1) years. This corresponded to a Kaplan-Meier survival of 97.4% (95% CI: 95.1–99.7) for the endpoint “implant revision” at 5 years (Fig. 3). Eight patients (4.0%) died from causes unrelated to their UKA, with their implant in situ at the time of death (Online resource SI1 - Table 3).

**Table 3:** Patient characteristics by Oxford knee score 12 months postoperatively

Group	OKS < 27	OKS $\:\ge\:$ 27	*P*-value
Patients (knees)	27	147	
Number of males, N (%)	11 (40.7)	68 (45.2)	*0.75*
Mean age (years) (SD)	60.3 (12.8)	66.4 (9.3)	*0.026*
Mean body mass index (kg/m2) (SD)	31.3 (8.2)	30.4 (5.4)	*0.56*
*ASA score, N (%)*			
ASA 1	4 (14.8)	26 (17.7)	*0.72*
ASA 2	14 (51.9)	98 (66.7)	*0.13*
ASA 3	9 (33.3)	23 (15.6)	*0.04*
Mean follow-up (SD), years	5.9 (1.3)	5.86 (1.0)	

Characteristics for patients with a poor OKS < 27 and an OKS above $\:\ge\:$ 27, 12 months postoperatively. Continuous data are summarized as mean and standard deviation (SD). Categorical data are displayed as *n* (%). ASA score American Society of Anesthesiologists Physical Classification System

### Postoperative radiographic outcomes

The mean FTA decreased from 182.0° (SD 3.3) preoperatively to 178.0° (SD 2.7) postoperatively. Postoperative alignment was within recommended boundaries in the majority of cases (Table 4). Exceptions included the mean medial tibial implant underhang in the coronal view (–0.9 mm), and the mean posterior underhangs in the lateral view (–0.2 mm). No medial overhang of the tibial implant exceeding 2 mm was observed. However, posterior overhang of the tibial implant occurred in 17 cases (8.5%). In total, 173 patients (86.5%) had tibial implant valgus/varus within the acceptable ± 5° of neutral in the coronal plane.

**Table 4:** Radiographic data of postoperative alignment together with the criteria from Oxford TM unicompartmental knee manual.

Postoperative alignment	Mean (SD)	Radiographic criteria
*Anterior-posterior evaluation*		
Anatomical femorotibial angle (FTA)	178° (2.7)	
Medial proximal tibial angle (MPTA)	84° (2.6)	87° ± 3
Outliers, *n (%)*	91/200 (45.5%)	
Femoral component varus/valgus (PAF)	-7.5° (3.6)	< 10° varus - < 10° valgus
Outliers, *n (%)*	33/200 (16.6%)	
Tibial component varus/valgus (PAT)	-2.9° (2.5)	< 5° varus - < 5° valgus
Outliers, n (%)	27/200 (13.5%)	
Medial tibial fit, overhang in mm (OM)	-0.9 (1.7)	Flush or < 2 mm overhang
>2 mm overhang, n (%)	0/200 (0%)	
*Lateral evaluation*		
Femoral component flexion/extension (PAF)	6.3° (3.4)	15° flexion+ or < 0° extension–
Outliers, *n (%)*	2/200 (1.0%)	
Posterior tibial slope (PAT)	3.5° (2.5)	7° + or – 5°
Outliers, *n (%)*	44/200 (22%)	
Femoral posterior overhang in mm (OF)	2.3 (1.5)	Flush or < 4 mm overhang
>4 mm overhang, n (%)	13/200 (6.5%)	
Anterior tibial underhang in mm (OTA)	-0.9 (1.8)	Flush or < 5 mm short
>5 mm underhang, n (%)	3/200 (1.5%)	
Posterior tibial overhang in mm (OTP)	-0.2 (2.0)	Flush or < 2 mm overhang
>2 mm overhang, n (%)	17/200 (8.5%)	

Categorical data are displayed as n (%). Continuous data are summarized as mean and standard deviation (SD). Alignment criteria are derived from Oxford ^TM^ unicompartmental knee surgical technique manual. *FTA* Femoral-shaft-tibial-shaft angle (anatomic angle), *MPTA* medial proximal tibial angle, *PAF* femoral component varus/valgus, *PAT* tibial component varus/valgus, *OM* medial tibial overhang, *OF* femoral posterior overhang, *OTA* anterior tibial underhang, *OTP* posterior tibial overhang [18] .

## Discussion

This study evaluates the learning curve associated with the implementation of mUKA, distinguishing between learning in operative efficiency and learning related to patient safety. Formal learning-curve analyses were applied to surgical duration, as this process measure is expected to change early with increasing experience and is more directly related to operative performance. Using this approach, a reduction in duration of surgery was observed after 55 cases. In contrast, patient reported outcomes and survival were evaluated across the full study period to assess safety and clinical effectiveness, allowing efficiency gains during implementation to be assessed while ensuring continuous monitoring of patient-centred outcomes and implant survival. The observed transition after 55 cases should be interpreted as a stabilization of the operative process at an institutional level rather than a benchmark of surgical speed alone. While previous UKA learning curve studies have reported learning effects after approximately 25–50 cases [14, 24, 25], most have defined learning based on revision rates or implant positioning [10, 24], outcomes that reflect different aspects of performance than process-based measures such as surgical duration. PROMs remained stable throughout the implementation phase. The stability of PROMs despite ongoing improvements in operative efficiency may be explained by the surgeons’ substantial prior experience with knee arthroplasty, which may have ensured a high and consistent level of surgical safety from the outset of mUKA implementation. These findings indicate that improvements in operative efficiency can be achieved during early mUKA implementation without adverse effects on clinical outcomes. In the final segment of the CUSUM curve, a slight increase in duration of surgery was observed, likely reflecting increased surgeon confidence and a gradual shift toward more complex cases. As proficiency develops, surgeons may feel more comfortable undertaking technically demanding procedures, which in turn contributes to the observed variation in duration of surgery, a pattern that has been reported in other procedural learning curves [26, 27]. A comparison of PROMs between patients with excellent and poor outcomes revealed notable demographic differences. Patients with poor OKS were more frequently younger and had higher ASA scores. Younger age has previously been associated with inferior patient-reported outcomes and higher failure rates following UKA [28, 29]. Importantly, this association may in part reflect expectation bias, as younger patients often report higher functional demands and activity expectations that may exceed the achievable performance of a unicompartmental implant [30]. As a result, lower PROM scores in younger patients may not necessarily indicate inferior surgical performance, but rather differences in patient expectations and outcome perception. Similarly, increased ASA scores have been associated with poorer functional outcomes, which aligns with the trends observed in our cohort [22, 23]. The revision rate of 4.0% over a mean follow-up of 5.8 years aligns with the lower end of rates reported in the literature [31–34], which range from 3.4% to 13%. National registry data from Denmark (2020) indicate a 6.6% revision rate for primary mUKA after five years [35], suggesting that our outcomes are comparable to the national standard. Furthermore, our study’s five-year implant survival rate of 97.4% is consistent with previously published survivorship data (84–98%) [36–40], further reinforcing the safety and effectiveness of mUKA in clinical practice. Postoperative radiographic analysis indicates a tendency toward tibial component undersizing. This likely reflects a deliberate surgical choice influenced by research suggesting an association between tibial oversizing and postoperative pain or functional limitations [41, 42]. Importantly, no revisions were required due to tibial loosening, supporting the notion that minor tibial underhang does not necessarily compromise implant longevity. A previous study [42] has similarly demonstrated that tibial underhang of up to 3 mm does not increase revision risk, further validating our findings. The mean posterior tibial slope was slightly lower than the intended target of 7°, but remained within the predefined acceptable range. This study has several limitations. Its single-center design might have limited generalizability, and both participating surgeons had prior TKA experience, which might not fully reflect the learning curve of less experienced or low-volume surgeons and centers. Additionally, our response rate of 87% introduces potential selection bias, as non-responders were significantly younger than responders. Furthermore, the use of short-leg radiographs instead of full-leg radiographs, together with variation in radiographic image quality, represents a limitation for alignment interpretation and may have introduced measurement inaccuracies. When interpreting comparisons with national registry data, a small degree of reporting inaccuracy cannot be entirely excluded. However, as analyses were restricted to revision endpoints, this is unlikely to affect the present findings. Despite these limitations, the study benefits from a homogeneous patient cohort with isolated anteromedial osteoarthritis and a high PROM response rate, enhancing its internal validity and supporting the robustness of its conclusions regarding the clinical efficacy of mUKA during its implementation phase.

### In conclusion

The implementation of mUKA in this setting was associated with favourable clinical outcomes, with no compromise observed in PROMs. Duration of surgery decreased after 55 cases, while PROMs remained stable, indicating favorable outcomes regardless of the learning curve. High surgical precision was maintained even during the early learning phase. Within the context of surgeons with substantial prior experience in knee arthroplasty, mUKA appears to be a reliable treatment modality for eligible patients during its initial implementation phase. These findings may provide a practical reference for centers planning mUKA implementation.

## Supplementary Information

Below is the link to the electronic supplementary material.

Supplementary Material 1

Supplementary Material 2

## Data Availability

The datasets generated and analyzed during the current study are not publicly available due to Danish data protection regulations but are available from the corresponding author upon reasonable request and with permission from the Danish Data Protection Agency (approval no. P-2022-290).
