# Supplementary material for: Starting up a cementless Oxford medial unicompartmental knee arthroplasty practice: a prospective cohort study of 200 knees
Source: Arch Orthop Trauma Surg. 2026 Feb 26;146(1):86. doi: 10.1007/s00402-026-06229-z (PMC12946369; doi:10.1007/s00402-026-06229-z)
Supplement: Supplementary file 1 — Supplementary Material 1 [file 402_2026_6229_MOESM1_ESM.pdf]

Supplementary information 1 (SI1)
Title: Starting up a Cementless Oxford Medial Unicompartmental Knee Arthroplasty Practice - A Prospective Cohort Study of 200 Knees
Journal: Archives of Orthopaedic and Trauma Surgery

Table 1: Demographic comparison of patients with and without a completed 12-months Oxford Knee Score

Group	Complete 12 months OKS	Missing 12 months OKS
**Patients (knees)**	174 (87%)	26 (13%)
**Number of males, n (%)**	77 (44.3)	10 (38.5)
**Mean age (years) (SD)**	65.5 (10.0)	60.4 (11.6)
**Mean body mass index (kg/m2) (SD)**	30.5 (5.8)*	32.4 (7.5)
**ASA score (%)**		
**- ASA 1**	30 (17.2)	3 (11.5)
**- ASA 2**	113 (64.9)	18 (69.2)
**- ASA 3**	31 (17.8)	5 (19.2)
**Mean follow-up (SD)**	5.9 (1.1)	5.7 (1.6)

n =172

Categorical data are displayed as n (%). Continuous data are summarized as mean and standard deviation (SD). BMI = body mass index (kg/m^2). ASA score: American Society of Anesthesiologists Physical Classification System.

Table 2: Patient reported outcome measures postoperatively.

Median (IQR)	OKS	APQ	FJS
3 months	36 (28-40), n=177	44 (22-69), n=179	52 (34-68), n=178
12 months	41 (34-44), n=174	66 (32.5-88), n=175	65 (46-83), n=174
24 months	42 (35-45), n=166	70.5 (38-97), n=166	69 (44-88), n=165

Continuous data are summarized as median and interquartile range (IQR). OKS: Oxford Knee Score, APQ: Activity & Participation Questionnaire, FJS: Forgotten Joint Score. *n* represents the number of patient responses.

Table 3: Overview of causes and surgical interventions for reoperation and revision

**Re-operations, revisions, deaths**				
**Category**	**Causes**	**Procedure**	**n**	**Case number**
Reoperation	Progression of OA	Lateral UKA	3	7, 74, 157
	Reduced range of motion	MUA	1	44
	Impingement	Arthroscopic debridement	2	82,197
			Total = 6	
Revision	Progression of OA	TKA	5	19, 21, 48,91,121
	Pain	TKA	1	52
	Bearing dislocation	Bearing exchange	2	88, 108
			Total = 8	
Death	*Cause unrelated to the UKA*		8	
			Total = 8	

Categorical data are displayed as n. OA: Osteoarthritis. UKA: unicompartmental knee arthroplasty.

TKA: total knee arthroplasty. MUA: Manipulation under anesthesia

Figure 1: Distribution of revision and reoperation events along the learning curve (CUSUM)

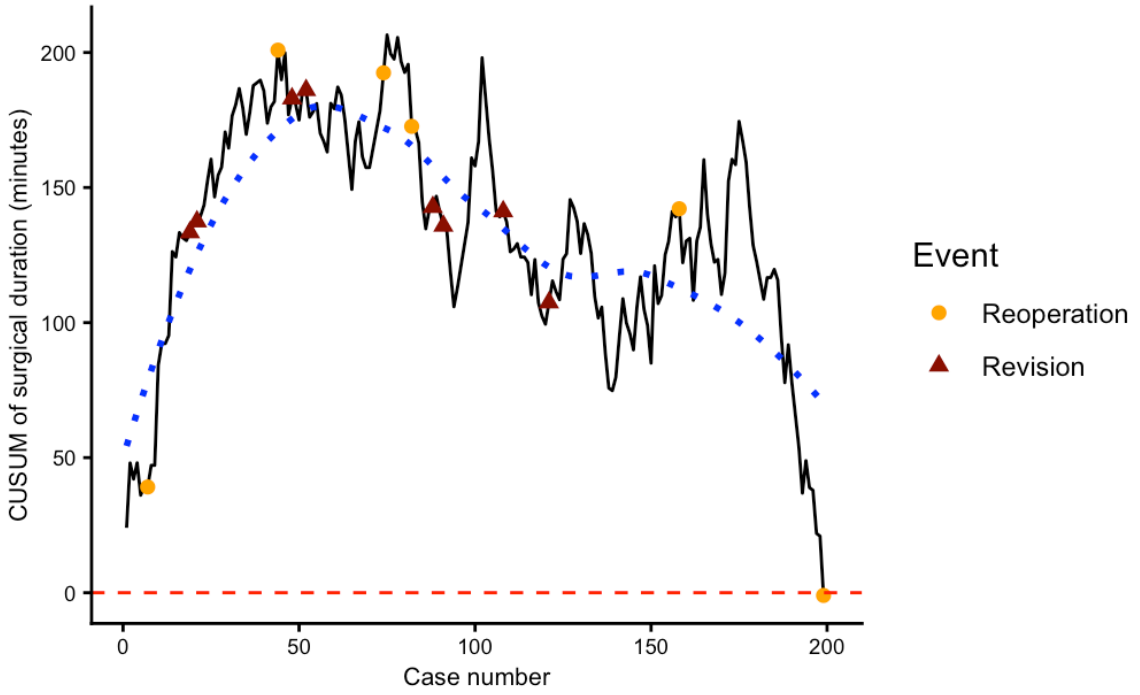

Figure 1: The figure illustrates the cumulative sum (CUSUM) of surgical duration across consecutive cases, with revision and reoperation events superimposed on the learning curve. The x-axis represents the chronological case number and the y-axis the CUSUM of surgical duration. Revision events are indicated by triangles, and reoperation events by circles. Revision events were predominantly observed during the early to mid-phase of the learning curve, whereas reoperations occurred throughout the case series, including the late phase.
