# Supplementary material for: Starting up a cementless Oxford medial unicompartmental knee arthroplasty practice: a prospective cohort study of 200 knees
Source: Arch Orthop Trauma Surg. 2026 Feb 26;146(1):86. doi: 10.1007/s00402-026-06229-z (PMC12946369; doi:10.1007/s00402-026-06229-z)

Supplementary information 2 (SI2)
Title: Starting up a Cementless Oxford Medial Unicompartmental Knee Arthroplasty Practice - A Prospective Cohort Study of 200 Knees
Journal: Archives of Orthopaedic and Trauma Surgery

Radiographic overview

Preoperative Radiographic Alignment

Blue lines serve as visual guides; green lines represent actual measurements.

Anterior-Posterior View (APV)

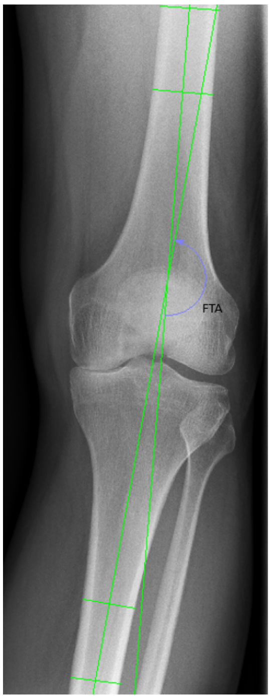

Femorotibial Angle (FTA)

• Measured as the angle between the anatomical axes of the femur and tibia.

• Each axis is constructed by connecting two mid-diaphyseal points along the femoral and tibial shafts. Lines are drawn through the midpoints on each bone.

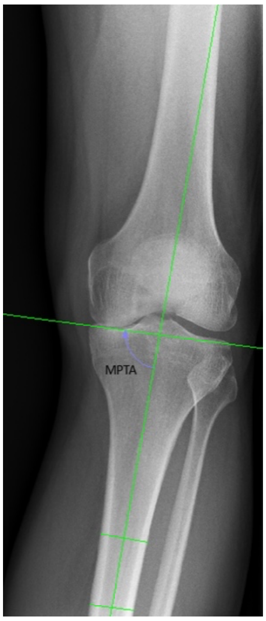

Medial Proximal Tibial Angle (MPTA)

• Defined as the angle between the anatomical axis of the tibia and the tibial plateau.

• The tibial axis is drawn using two mid-diaphyseal points.

• The tibia plateau line is drawn between the deepest points of the medial and lateral tibial condyles.

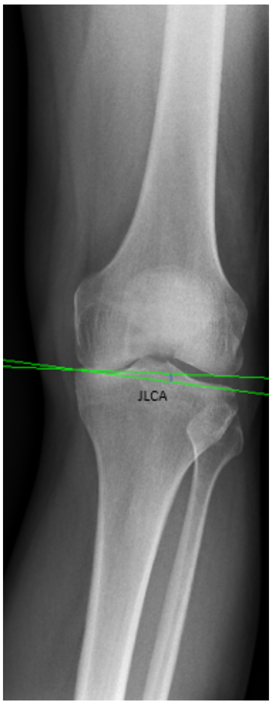

Joint Line Convergence Angle (JLCA)

• Measured as the angle formed between two tangential lines drawn along the distal medial and lateral femoral condyles and the tibial plateau.

Lateral View

Posterior Tibial Slope (PTS)

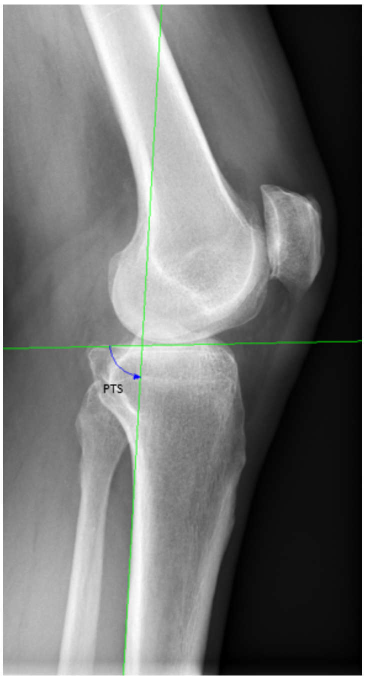

• Measured as the angle between the tibial plateau and the longitudinal axis of the tibia.

• The tibial axis is drawn along the posterior cortex, while a second line is drawn along the slope of the tibial plateau.

Bone-on-Bone Contact

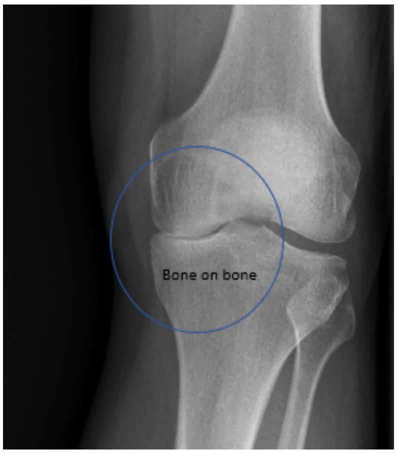

• Evaluated on the AP view to confirm complete loss of medial joint space.

• This finding served as an inclusion criterion for the Oxford Partial Knee Replacement, indicating advanced osteoarthritis of the medial compartment.

<u>Postoperative Radiographic Alignment</u>

Anterior-Posterior View (AP)

Femorotibial Angle (FTA)

• As previously described, used to evaluate postoperative correction of the axis.

• The anatomical axes of femur and tibia are constructed by connecting two mid-diaphyseal points located approximately at one-third and two-thirds of the visible shaft length for each bone.

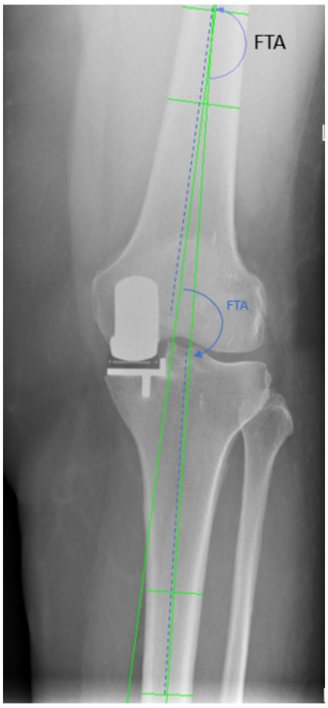

Medial Proximal Tibial Angle (MPTA)

• Assesses the coronal orientation of the tibial component relative to the tibial anatomical axis.

• The tibial axis is defined as above.

• The articular surface of the tibial component is represented by a line drawn along the superior edge of the baseplate.

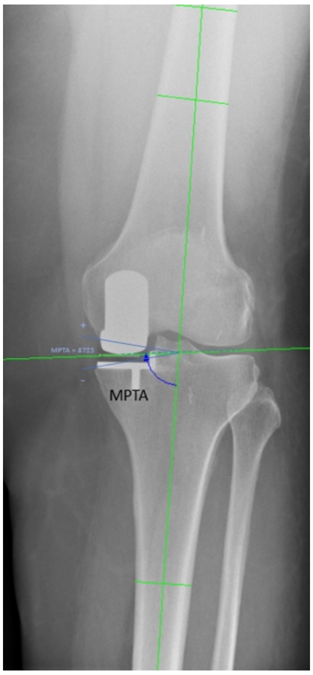

Femoral Component Varus/Valgus Angle (PAF)

• Describes the coronal alignment of the femoral component relative to the femoral anatomical axis.

• The femoral axis is drawn as described above.

• A secondary axis for the femoral component is established by connecting two midpoints along the component's body, typically at its proximal and distal regions.

• The angle is measured between the anatomical femoral axis and the axis of the femoral component, reflecting the degree of varus or valgus alignment of the femoral component. .

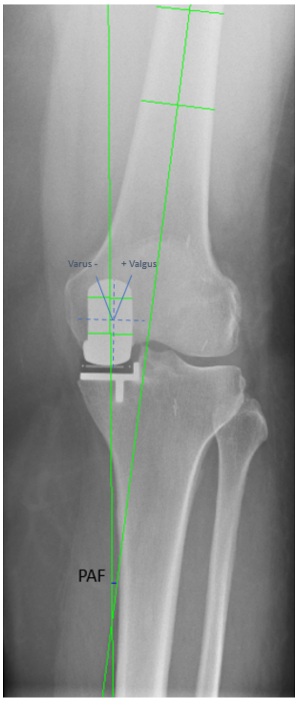

Tibial Component Varus/Valgus Angle (PAT)

Describes the coronal alignment of the tibial component relative to the tibial anatomical axis.

• The tibial axis is drawn as described above.

• The tibial component´s orientation is represented by a line along its inferior surface.

• The angle is measured between the anatomical tibial axis and the axis of the tibial component, reflecting varus or valgus alignment of the tibial component.

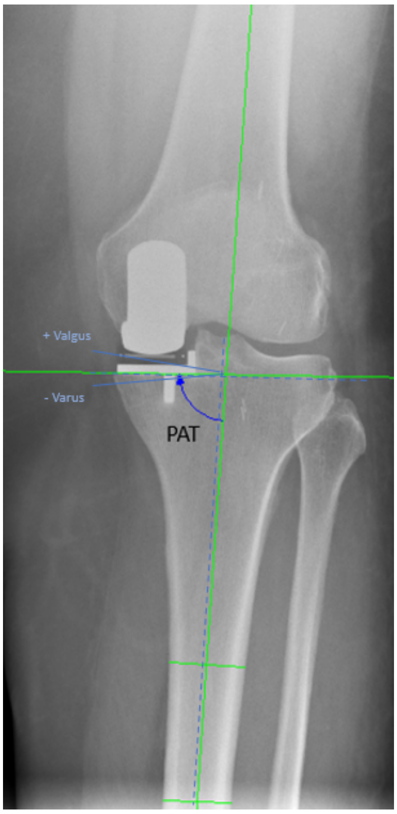

Medial Tibial Overhang/Underhang (OM)

• Measures the horizontal offset between the medial edge of the tibial component and the cortical margin of the proximal tibia.

• Measured in millimeters using a perpendicular line from the medial edge of the component to the tibial cortex.

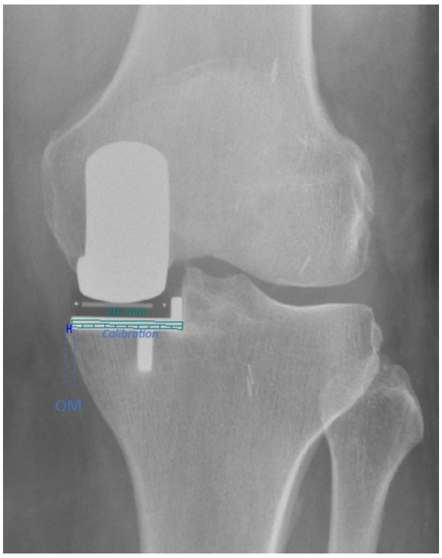

Lateral View

Femoral Component Flexion/Extension Angle (PAF)

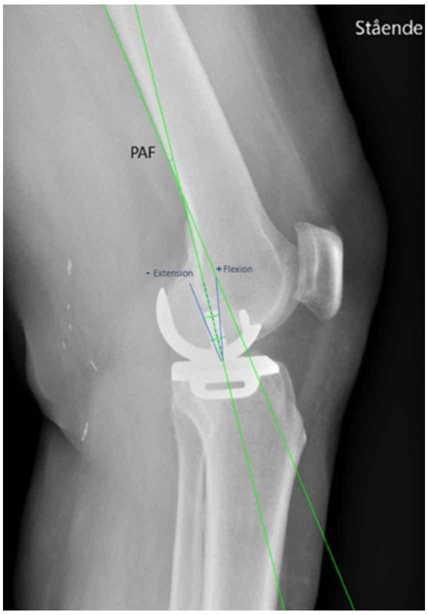

- Assesses the lateral alignment of the femoral component relative to the femoral shaft.
- The femoral axis is defined by drawing a line along the posterior cortex of the femoral shaft.
- The orientation of the femoral component is defined by drawing a central axis through the component's body, created by connecting the midpoints of two transverse lines—typically one at the proximal and one at the distal end of the femoral component.
- The angle between these two lines reflects flexion or extension of the femoral implant.

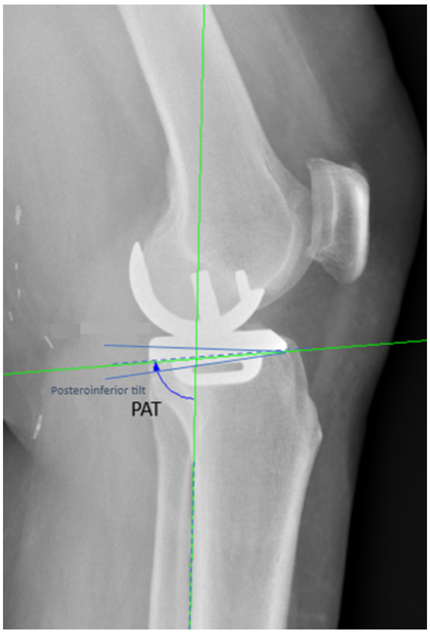

Posterior Tibial Slope (PAT)

- Measured as the angle between the surface of the tibial component and the longitudinal axis of the tibia in the lateral plane.
- The tibial axis is drawn along the posterior cortex of the tibial shaft
- A second line is drawn along the horizontal surface of the tibial component.

Component Overhang/underhang Measurements - Femoral Posterior Overhang **(OF)**, Anterior Tibial underhang **(OAT)**, and Posterior Tibial Overhang **(OTP)** are each measured in millimeters.

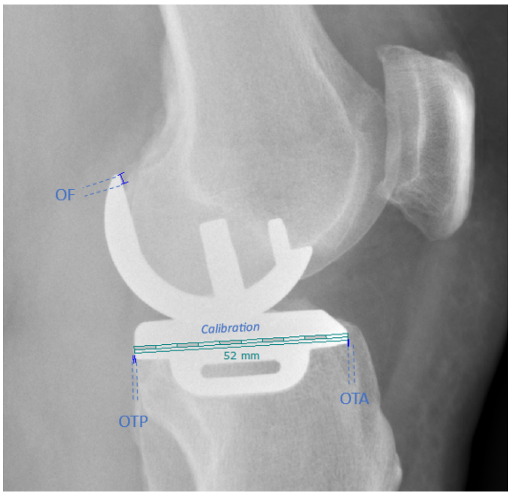

- Defined as the distance between the edge of the component and the edge of the adjacent bone surface.
- A positive value indicates overhang; a negative value indicates underhang.

Radiographic measurements overview:

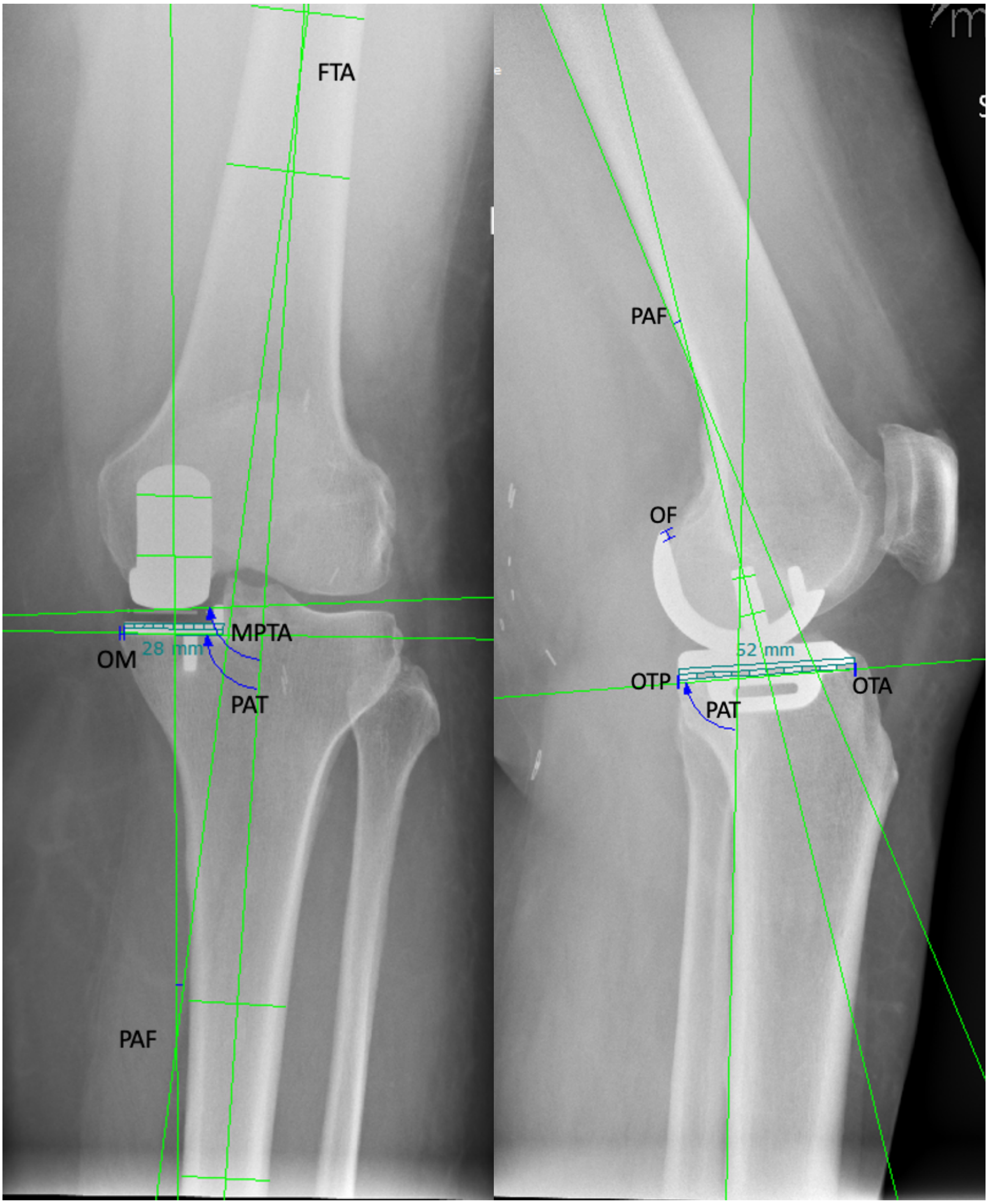

Supplement: Supplementary file 2 — Supplementary Material 2 [file 402_2026_6229_MOESM2_ESM.pdf]
